# The ultra-early protective effect of ulinastatin on rabbit acute lung injury induced by paraquat

**DOI:** 10.1186/1471-227X-13-S1-S7

**Published:** 2013-07-04

**Authors:** Zujun Song, Gaofei Chen, Gang Lin, Chiyu Jia, Jianxia Cao, Guokun Ao

**Affiliations:** 1 The Emergency Department, The People's Liberation Army No. 309 Hospital, Beijing, China; 2 the Graduate School of the Fourth Military Medical University, Beijing, China; 3 the Chinese People's Liberation Army No. 309 Hospital of Burns and Plastic Surgery, Beijing, China; 4 the Chinese People's Liberation Army No. 309 Hospital of Radiology, Beijing, China

## Abstract

**Objective:**

To study ultra-early pathophysiological changes of rabbit acute lung injury (ALI) caused by paraquat (PQ) and discuss the ultra-early protective effect of ulinastatin on rabbit ALI due to PQ.

**Methods:**

30 New Zealand white rabbits were randomly divided into a control group, a paraquat group and an ulinastatin intervention group with 10 rabbits in each group. For paraquat group and intervention group a single dose of paraquat (35mg/kg) was injected intraperitoneally to establish rabbit models of ALI. The control group was injected an equal volume of saline. The intervention group was treated with 100Ku/kg ulinastatin immediately after the establishment of the ALI model. The respective experimental groups underwent 320-slice CT perfusion scan of pleural at 2h, 4h and 6h time point after modeling to get CTP (CT Perfusion) images and related parameters. 2mL blood was collected in the marginal ear vein to determine the mass concentration of the vascular endothelial growth factor (VEGF). The animals were killed by air embolism after 6h and lung tissue was taken for pathology observation.

**Results:**

The reginal blood flow (rBF) and reginal blood volume (rBV) of paraquat group at 2,4,6 h time point were significantly (P <0.05) lower than those of control group. The intervention group rBF and rBV at 2, 4 and 6 h time points were significantly higher (P <0.05) compared to paraquat group. The permeability surface (rPS) and VEGF mass concentration of paraquat group at 2,4,6 h time point were significantly higher than the control group (P <0.05), and the intervention group rPS and VEGF mass concentrations at 2,4,6h time point were significantly lower (P <0.05) than those of paraquat group. Pathological detection indicators of paraquat group (congestive capillary percentage, the number of red blood cells outside of capillaries, percentage of capillaries with basement membrane damage) were significantly higher (P <0.05) at 6h time point compared with the control group, while significantly lower (P <0.05) in intervention group than in paraquat groups. Pathological observation under light microscope showed in paraquat group obvious inflammatory cell infiltration, alveolar epithelial cell hyperplasia, widened alveolar septum, visible focal hemorrhage, visible acute and chronic inflammatory cell infiltration in bronchioles cavity; under electron microscopy alveolar epithelial cell degeneration and necrosis, vascular welling of the endothelial cells, basement membrane rupture, a lot of exudates in alveolar space. In the intervention group, the above the symptoms were mitigated.

**Conclusion:**

In the ultra-early stage of rabbit ALI induced by PQ, pulmonary vascular endothelial cell is damaged and serum VEGF mass concentration and pulmonary vascular permeability increase. Early ulinastatin intervention can reduce serum VEGF level and PQ-induced vascular permeability amplitude, indicating that ulinastatin has a protective effect on pulmonary vascular endothelial cells.

## Introduction

Acute lung injury (ALI) is acute progressive dyspnea and refractory hypoxemia caused by a variety of lung internal and external factors. The basic pathological changes are acute inflammatory response and increase of pulmonary microvascular permeability. Ulinastatin is an urinary trypsin inhibitor isolated from male urine and it can inhibit the release of a variety of inflammatory mediators. In recent years, ulinastatin was widely used in the treatment of a variety of severe diseases[1]. It has been reported that early application of ulinastatin can reduce the symptoms of ALI [2], but the mechanism is not fully elucidated. 320-Slice CT is the world's most advanced imaging equipment, and is currently the only CT that can achieve dynamic volume scan imaging with functional imaging characteristics. This article intents to explore the protective effect of ulinastatin on lungs at ultra-early ALI stage and provide the experimental basis for clinical treatment through establishment of rabbit ALI model by paraquat poisoning and 320-slice CT imaging technology to observe diseased lung function status and blood flow situation as well as changes in the concentration of serum VEGF and pathological test results.

## Material and methods

### Experimental material

Paraquat aqueous solution was purchased from Chuandong Agrochemical Co., Ltd. (active ingredient content of 200g / L, the production license number: XK13-003-00058); 320-slice CT was purchased from Toshiba Corporation, Japan; VEGF kit was purchased from Beijing Dongge reagents Limited; Optical microscope (CX31RTSF type) was purchased from Olympus Corporation; Electron microscopy equipment was provided by the fourth Military Medical University, Institute of Neurobiology.

### Animal grouping

Thirty healthy New Zealand white rabbits, 3-6 months of age, body weight between 2.0-2.5kg, both male and female, were provided by the Beijing Haidian District Xinglong experimental animal breeding plant, the Certificate No. SCXK (Beijing) 2010-0011. The animals were randomly divided into three groups: control group, paraquat group, ulinastatin intervention group, with ten rabbits in each group.

### Model preparation

Referenced by Piao Zhiyong et al [3] rabbit ALI modeling method, a single dose of 35mg/kg paraquat was intraperitoneally injected to paraquat group and ulinastatin intervention group respectively and the control group was injected an equal volume of saline instead. Animal behavior was observed to confirm successful modeling. In intervention group immediately after the establishment of the ALI model, 100Ku/kg ulinastatin was dissolved in 20 mL of saline for slow bolus in ear vein.

### Sample collection and indicator detection

Each experimental group underwent lipiodol perfusion (2ml) in the marginal ear vein after modeling 2,4 and 6 hours followed by immediate 320-slice CT scan of pleural. Images were acquired automatically and image analysis took middle and lower lung level CTP parameters from the same rabbit, avoiding the heart and diaphragm artifact interference. Blood (2ml) was collected at various time points in the marginal ear vein and centrifuged under 3000r/min for 10 minutes. The supernatant was used for VEGF mass concentration measurement with VEGF kit, strictly in accordance with the manual operation. After 6 hours animals were killed by air embolism and small piece of the right upper lobe tissue was taken for pathology observation under light and electron microscopy. Under light microscope, 10 fields were taken randomly for each sample at × 40 magnification vision. With image analysis software, the numbers of red blood cells outside the capillary were calculated and averages per field were taken. At × 10 magnification, 10 fields were randomly selected to calculate the percentage of congestive capillaries in the total number of capillaries within fields. Under electron microscope, percentages of capillaries with damaged basement membrane in the total number of capillaries in the same sample were calculated.

### Statistical methods

SPSS 14.0 statistical software was used for experimental data analysis and measurement data was presented as mean ± standard deviation  form. One-way ANOVA was used for comparison between groups (LSD test for homogeneity of variance and Games-Howell test for arrhythmia) and P <0.05 indicates difference is statistically significant.

## Results

### Behavior change

Control group rabbits were full of energy, lively, breathing and heartbeat smooth; paraquat group of rabbits soon after exposure looked quiet without much movement, malaise, anorexia, shortness of breath, rapid heartbeat, and a few hours later showed lips cyanosis, twitching limbs and extrados; Ulinastatin intervention group of rabbit had relieved symptoms and did not appear cyanotic lips and convulsion.

### CTP parameter changes

**Figure 1:**
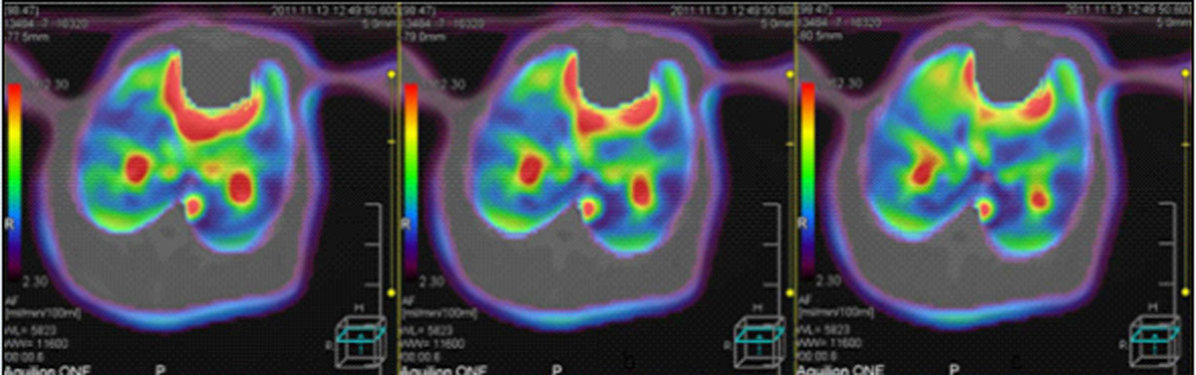
From left to right, CTP images of the same rabbit in control group at 2h, 4h, 6h time points, respectively. There were no significant changes, indicating good lung perfusion and no lung injury.

**Figure 2:**
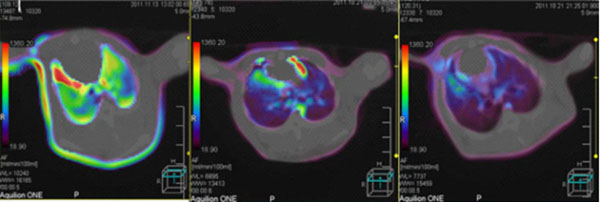
From left to right, CTP images of the same rabbit in paraquat group at 2h, 4h, 6h time points, respectively. The color changed most, indicating that pulmonary perfusion changed significantly over time, suggesting heavy lung tissue damage.

**Figure 3:**
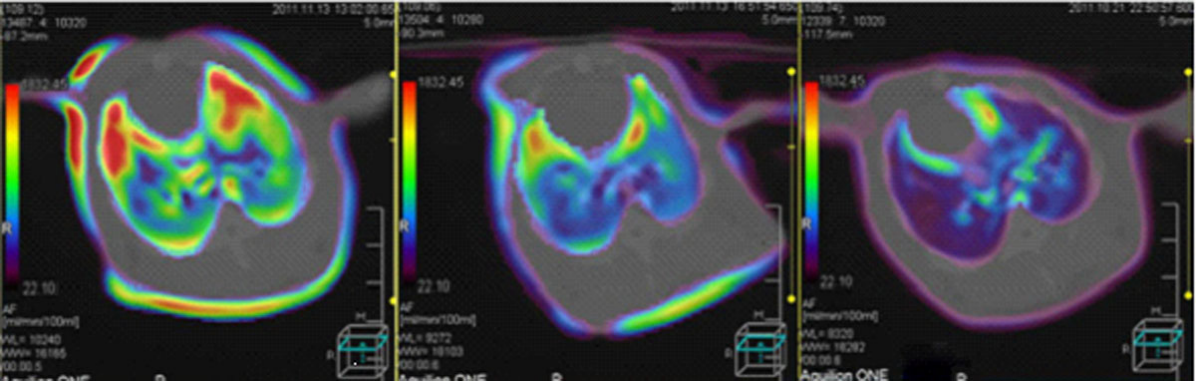
From left to right, CTP images of the same rabbit in Ulinastatin intervention group at 2h, 4h, 6h time points, respectively. The image changes were slightly smaller than paraquat group, indicating that the lung perfusion changed slightly, suggesting that lung injury was lighter.

For the same group of animals under the same parameter conditions, in the control group, perfusion images over time did not change significantly (Figure 1), indicating the lung perfusion in good condition. In paraquat group, perfusion image color changed the most (Figure 2), indicating that lung perfusion changed significantly over time, suggesting poor lung perfusion and heavy tissue damage. Ulinastatin intervention group image changes were slightly smaller than paraquat group (Figure 3), indicating that the lung perfusion changed slightly, suggesting that lung injury was lighter. Comparison of middle and lower lung level CTP parameters from the same rabbit showed that differences among paraquat group, control group and ulinastatin intervention group were statistically significant (P <0.05), shown in Table 1.

**Table 1:** CTP parameter changes of each experimental group at different time points

CTPparameter	Group	2h	4h	6h
rBF	control group	156.16±23.61	155.35±24.14	155.24±24.48
	paraquat group	120.77±12.99^*^	96.99±12.38^*^	66.98±11.05^*^
	intervention group	136.8±13.81^▲^	112.24±12.73^▲^	82.52±9.90^▲^
rBV	control group	14.25±1.75	14.32±1.70	14.56±1.91
	paraquat group	12.4±1.08^*^	10.72±0.91^*^	7.99±0.57^*^
	intervention group	13.57±1.13^▲^	12.16±0.99^▲^	9.60±1.49^▲^
rPS	control group	21.27±1.28	21.28±1.44	21.77±1.69
	paraquat group	31.14±2.53^*^	35.04±2.69^*^	38.82±2.57^*^
	intervention group	27.27 ±1.07^▲^	31.17 ±1.40^▲^	34.80±1.06^▲^

Note: At the same time point paraquat group compared with the control group * P <0.05, the intervention group compared with paraquat group ^▲^P<0.05.

(unit, rBF: ml/min/100ml,rBV:ml/100ml,rPS:ml/min/100ml)

### Changes in serum VEGF

Compared with the control group, paraquat group serum VEGF mass concentrations at 2,4,6 h time point were significantly higher (P <0.05); Compared with paraquat group, ulinastatin intervention group serum VEGF mass concentrations at 2,4,6 h time point were significantly lower (P <0.05), as shown in Table 2.

**Table 2:** Comparison of serum VEGF mass concentrations of experimental groups at different time points

Group	n	2h	4h	6h
Control group	10	42.35**±**6.12	43.21±5.49	42.67±4.97
paraquat group	10	167.52±18.83^*^	257.98±20.62^*^	343.98±21.76^*^
intervention group	10	110.04±16.05^▲^	183.33±17.45^▲^	269.79±20.7^▲^

Note: At the same time point paraquat group compared with the control group * P <0.05, the intervention group compared with paraquat group ^▲^P<0.05.

### Pathology detection indicators

After 6 hours, pathology indicators became significantly abnormal. In paraquat group congestive capillary percentage, number of red blood cells outside of capillaries and percentage of capillaries with damaged basement membrane were significantly higher than those of the control group (P <0.05), while the above values of ulinastatin intervention group ​​increased slightly, with a significant difference (P <0.05) from paraquat group. The relevant indicator test results are shown in Table 3.

**Table 3:** Pathological test results of experimental groups at 6h time point

Group	Congestive capillary percentage	The number of red blood cells outside the capillary	The percentage of capillaries with damaged basement membrane
Control group	0.98±1.31	2.1±0.99	0
paraquat group	6.77±1.57^*^	25.1±5.34^*^	6.49±2.39^*^
intervention group	4.11±0.93^▲^	14.6±2.12^▲^	3.34±1.96^▲^

Note: At the same time point paraquat group compared with the control group * P <0.05, the intervention group compared with paraquat group ^▲^P<0.05.

## The pathological changes of the lung tissue

### Light microscope observation

**Figure 4:**
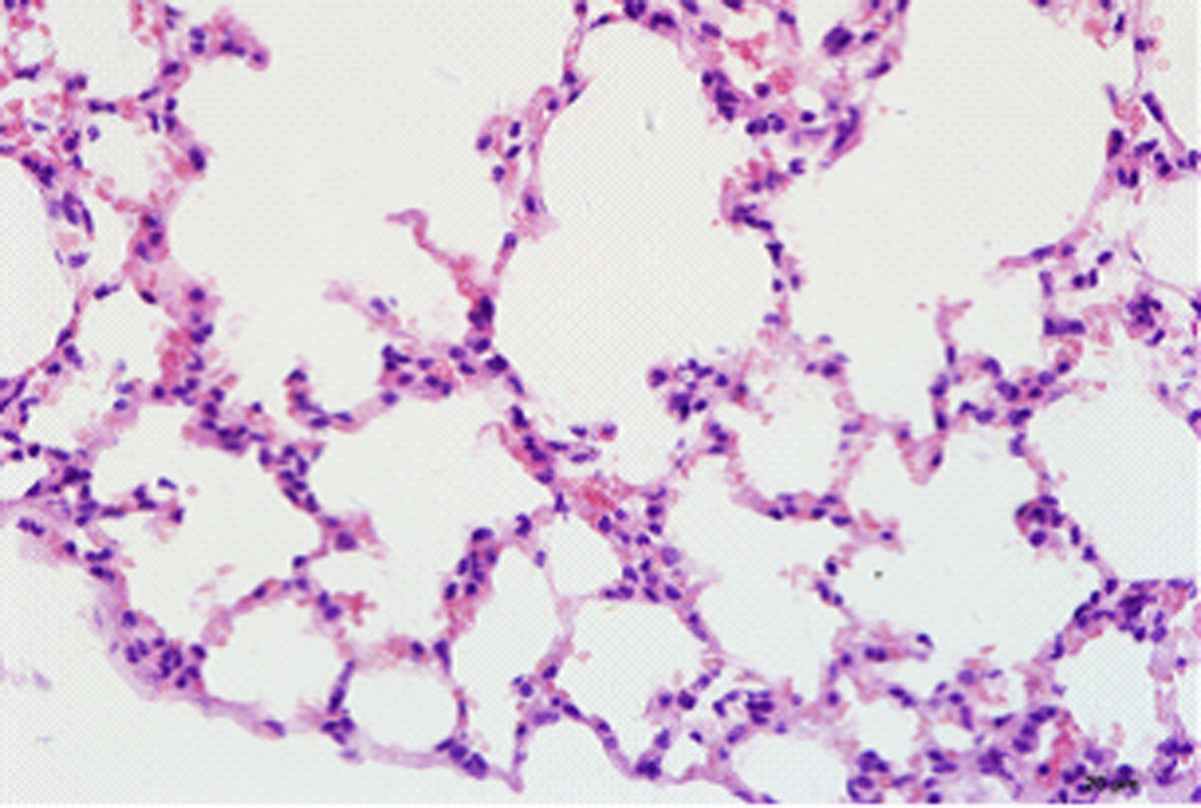
Control group animal’s lung tissue structure, alveolar structure clear, no inflammatory cell infiltration (HE×400)

**Figure 5:**
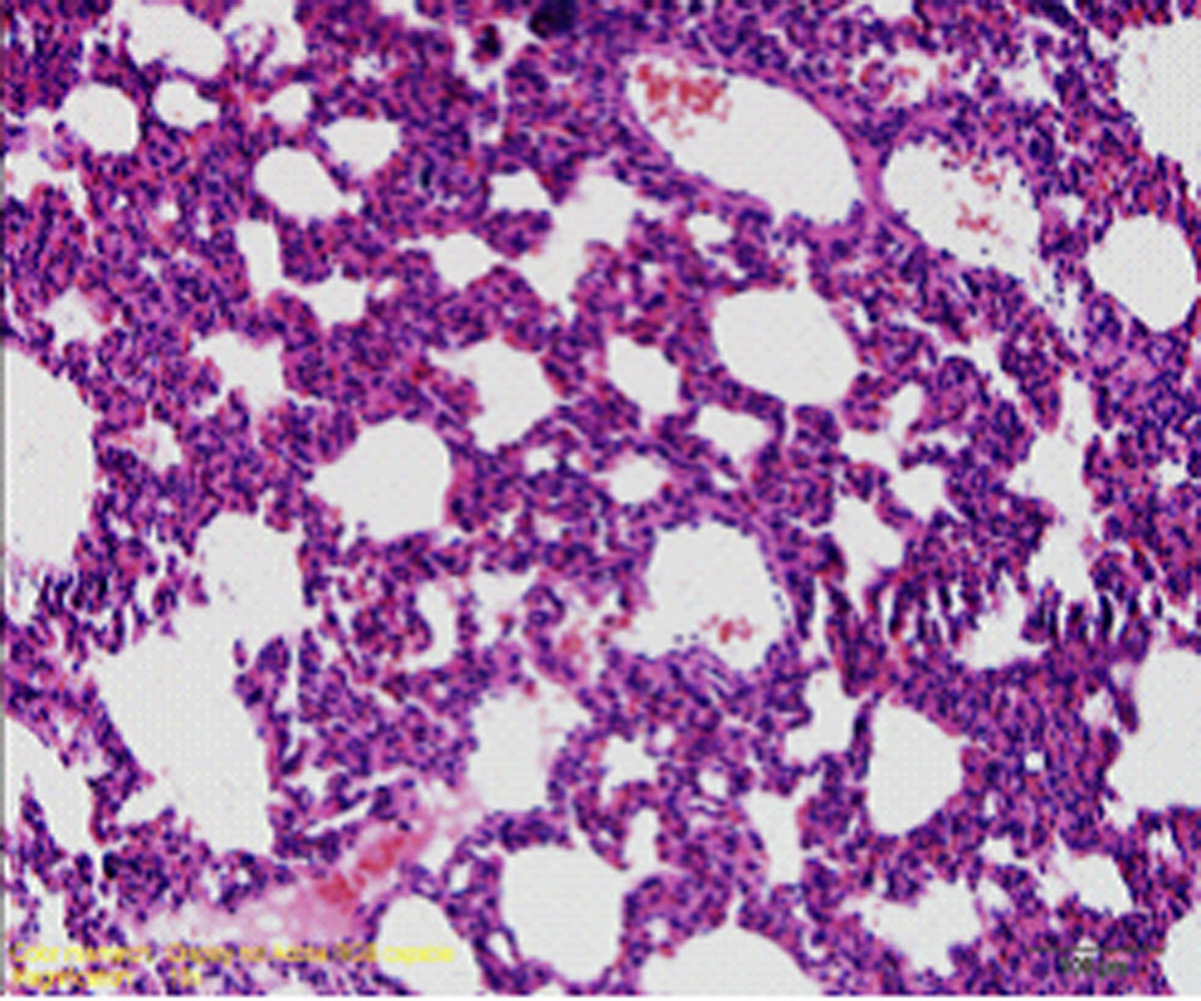
paraquat group animal’s lung tissue structure

**Figure 6:**
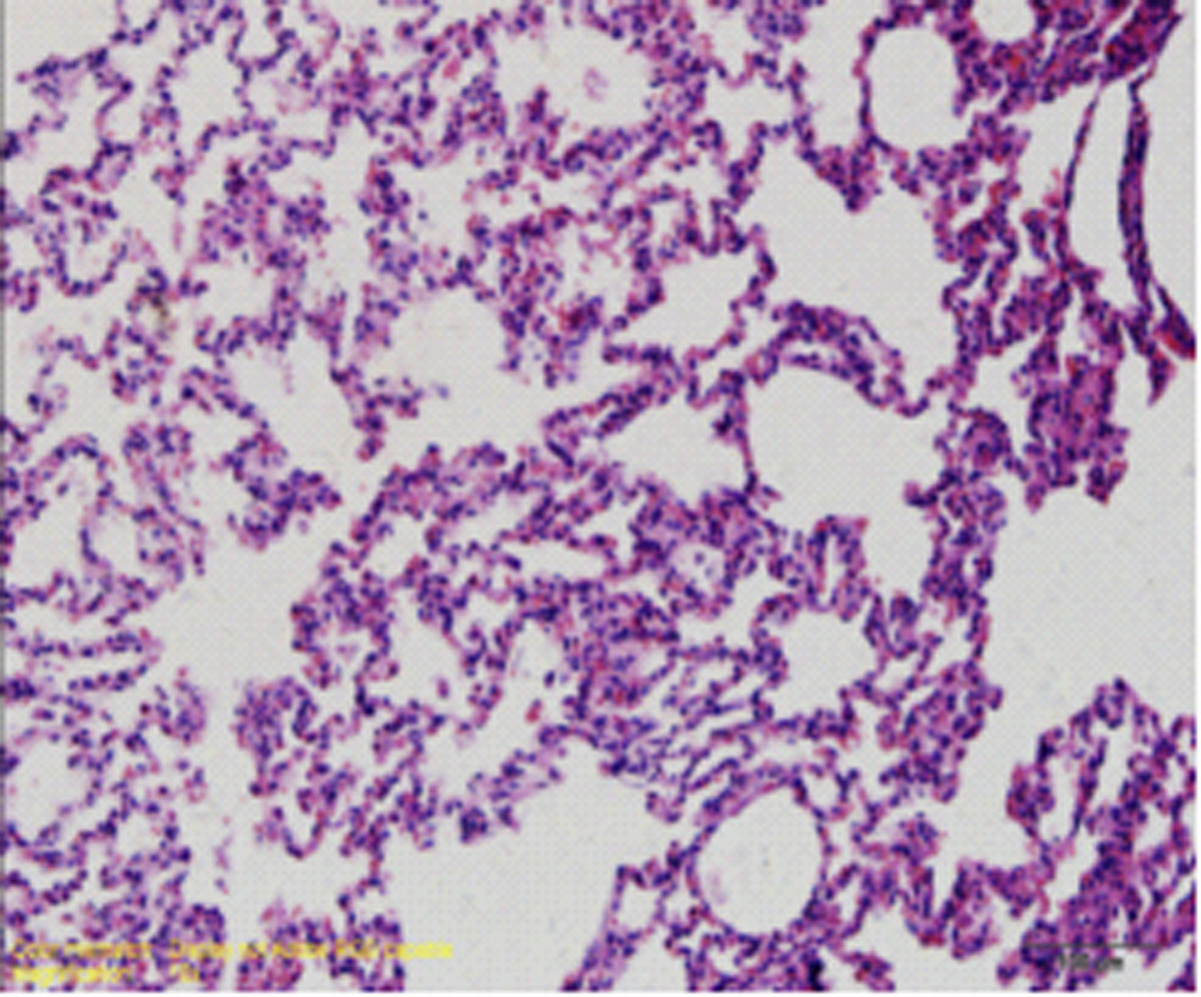
ulinastatin intervention group animal’s lung tissue structure a large number of inflammatory cell infiltration, focal hemorrhage (HE×400) no bleeding point (HE×400)

In control group, the basic structure of lung tissue was normal and alveolar structure was clear, no congestion, edema and inflammatory changes (Figure 4). In paraquat group, there was obvious lung tissue inflammatory cell infiltration, alveolar epithelial cell hyperplasia, widened alveolar septa, significant bronchiolar mucosal epithelial hyperplasia, spotty accumulation of lymphocytes, visible acute and chronic inflammatory cell infiltration within lumen, visible focal hemorrhage for part of lung tissue (Figure 5). Compared with paraquat group, the above symptoms in ulinastatin intervention group were reduced and there was no pulmonary hemorrhage (Figure 6).

### Electron microscope observation

**Figure 7:**
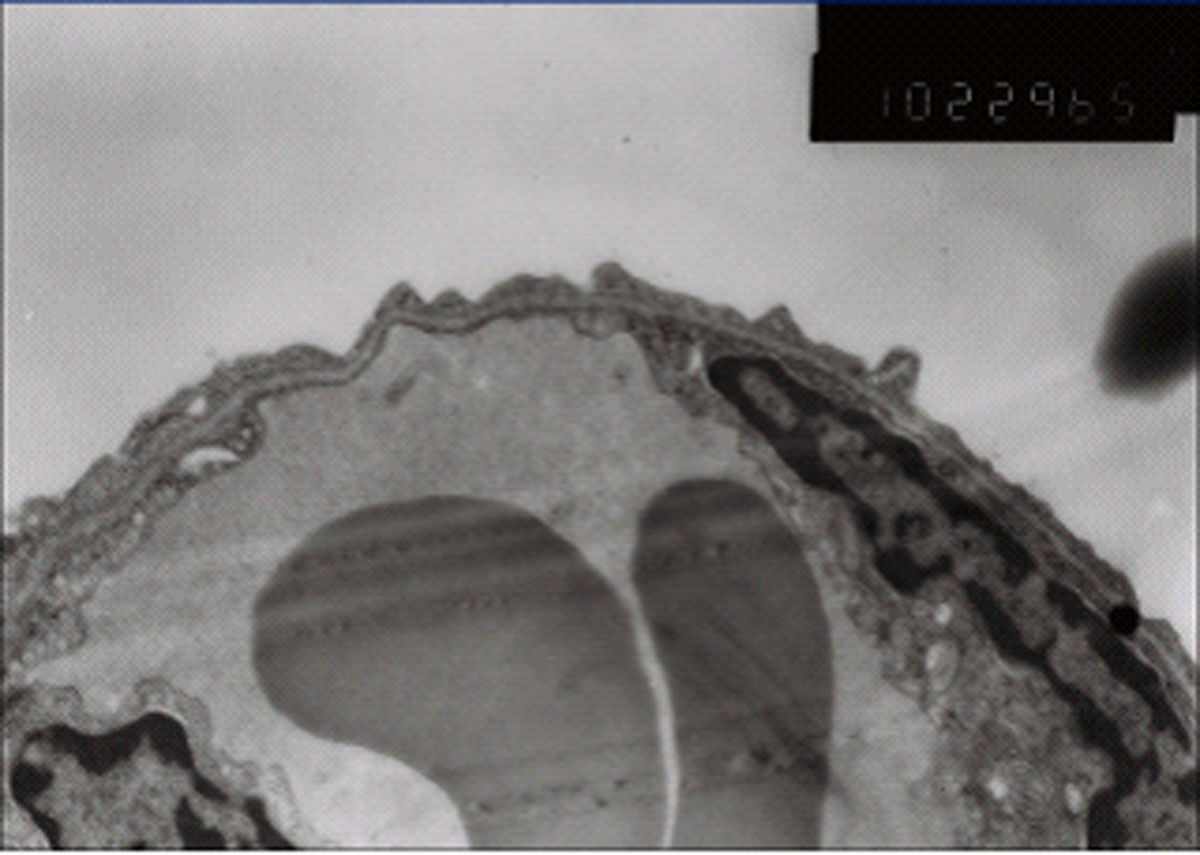
control group vascular basement membrane was complete, no swelling of endothelial cell (× 10K)

**Figure 8:**
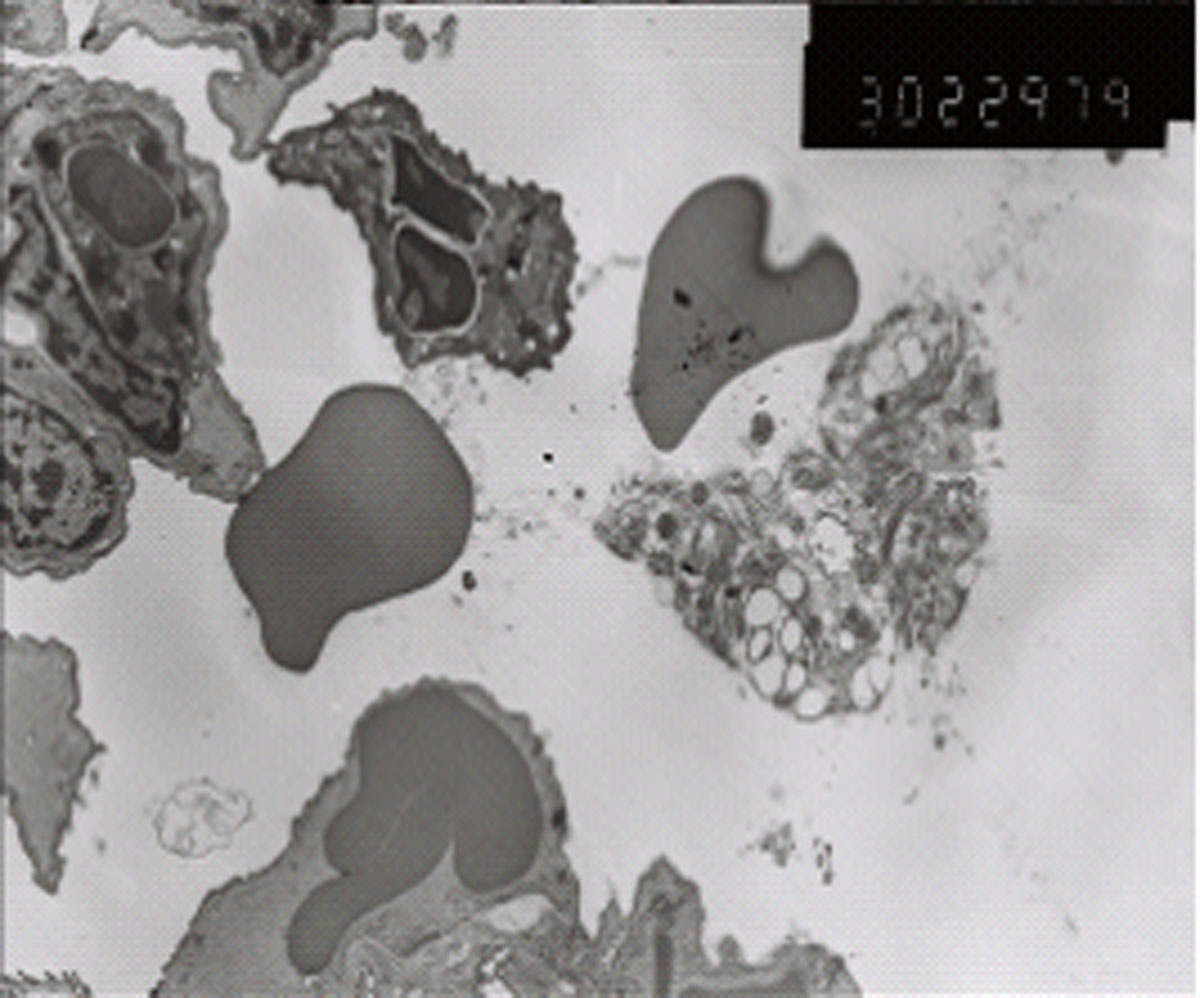
Paraquat group type I alveolar epithelial cell vacuolization structure

**Figure 9:**
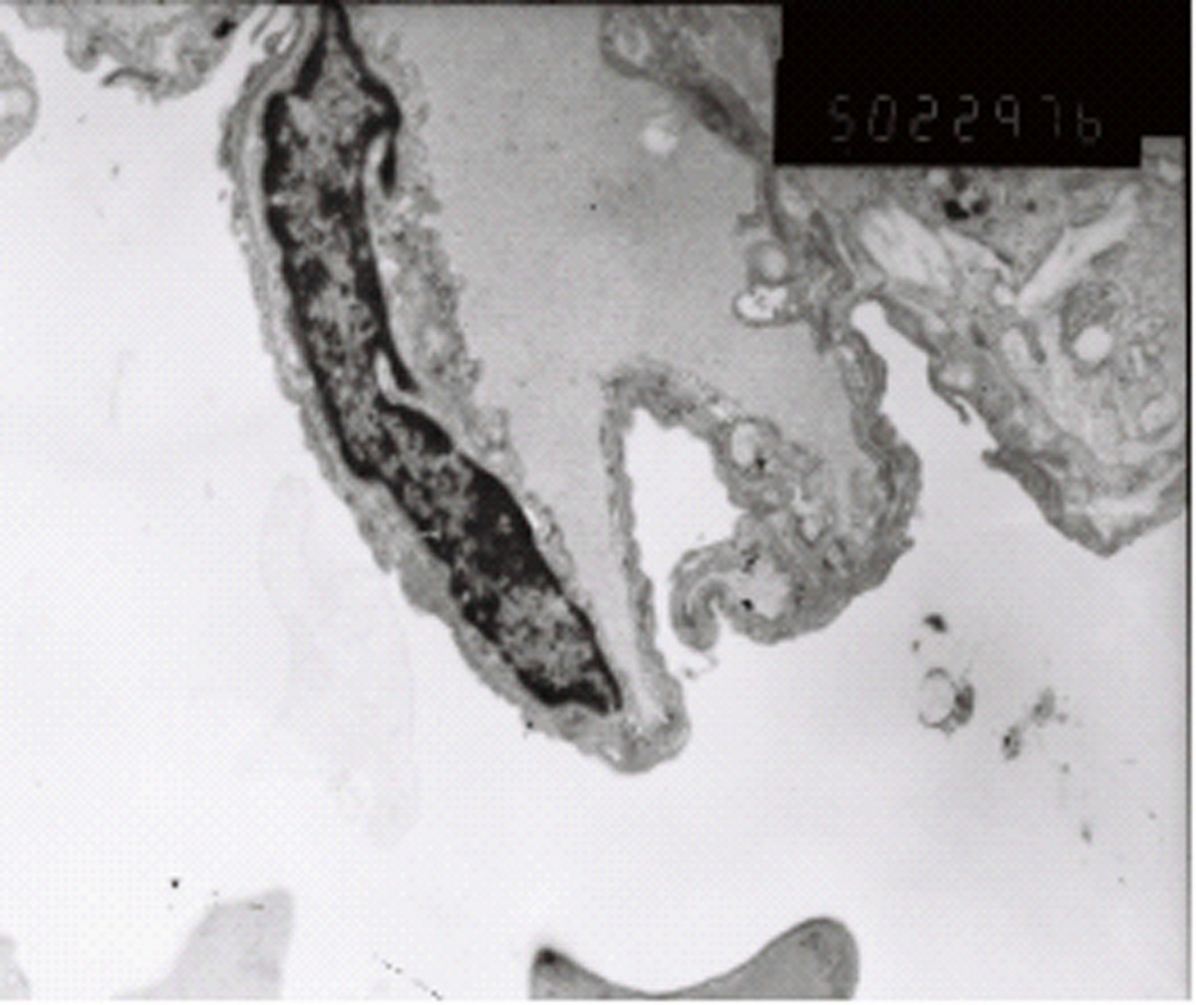
Ulinastatin intervention group endothelial cell swelling was not obvious, Neutrophils and red blood exudation in alveolar space。 (× 10K) Relative complete vascular basement membrane, a small amount of exudate in alveolar cavity (× 5K)

There was no abnormal change of ultrastructures in control group (Figure 7). The damage is obvious in paraquat group: type I alveolar epithelial cell vacuolization, degeneration and necrosis, type II alveolar epithelial cells swelling, obvious lamellar body emptying, large amount of microvilli shedding, a large number of vascular neutrophils, vascular endothelial cell swelling, basement membrane rupture, a large number of plasma-like exudates, red blood cells, granulocytes and necrotic cell debris in alveolar space (Figure 8). Ultrastructural changes of intervention group were smaller than those of paraquat group, vascular basement membrane was relatively complete, endothelial cell swelling was not obvious and exudate in alveolar cavity was less (Figure 9).

## Discussions

Acute lung injury (ALI) and its more severe stage of acute respiratory distress syndrome (ARDS) are caused by a variety of reasons both within and outside the lung characterized by progressive dyspnea and refractory hypoxemia. They are acute syndromes caused by body excessive inflammatory response. Endothelial cell damage and dysfunction are important pathological features of ALI / ARDS [4]. It is manifested as extensive damage of pulmonary vascular endothelial cells and alveolar epithelial as well as increase of pulmonary vascular permeability[5]. Ulinastatin is a urinary trypsin inhibitor isolated from male urine. It is a glycoprotein with typical Kuniz protease inhibitor structure. It has two completely non-overlapping active function areas and both have a broad spectrum of enzyme inhibition activity[6]. It has been confirmed that ulinastatin can simultaneously inhibit trypsin, hyaluronidase, elastase, phospholipase A2 and other varieties of hydrolytic enzymes [7]. It can also inhibit the release of inflammatory mediators and reduce the damage of inflammatory factor on target organs. Ulinastatin intervention of ALI is a research focus in recent years and studies have shown that ulinastatin can reduce symptoms of ALI, but its mechanism of action is unclear.

320-slice CT perfusion scan is a noninvasive functional imaging method [8]. CTP images were achieved by intravenous infusion of contrast agent and dynamic scanning to a particular level. Perfusion parameters such as rBF, rBV and rPS were obtained by computer processing and they can reflect hemodynamic changes in the capillary level to assess tissue and organ perfusion stage. In this paper, a single intraperitoneal injection of paraquat aqueous solution was used to establish ALI models and ulinastatin was used for ultra-early intervention. 320-Slice CT perfusion technology was applied for scan observation of blood flow changes in the early stages of ALI. Changes in serum VEGF levels and pathology detection indicators were combined to understand microvascular changes after ulinastatin intervention at ALI ultra-early stages and to explore early protective effect of ulinastatin on PQ-induced ALI in rabbits.

During experiments, we found that paraquat group animals appeared quiet without much movement, malaise, anorexia, shortness of breath, rapid heartbeat and other behavioral changes after exposure, in line with paraquat poisoning signs. Two, four, and six hours after exposure, lipiodol perfusion was followed by 320-slice CT scan of the chest. There was distinct color change of perfusion images with time in paraquat group, indicating that lung perfusion changed significantly over time, suggesting possible heavy lung tissue damages result in poor lung perfusion. Determination of CTP parameters found that in paraquat group rBF and rBV decreased with time, while rPS gradually increased with time. rBF reduction indicated blood rate declined in lung tissue; rBV reduction indicated blood capacity within the lung tissue vasculature decreased; rPS elevation suggested the rate of blood unidirectionally going into the tissue space through capillary endothelial cells increased. Three parameter values ​​at the same time point showed a significant difference (P <0.05) compared with the control group. The results verified the changes in the perfusion image, indicating poor lung perfusion at early ALI stage. This revealed ultra early hemodynamic characteristics of acute lung injury induced by paraquat. Ulinastatin group images showed little difference at the 2h time point from the control group, while significant difference at 4 and 6 hour time points. The former may be because paraquat absorption was small and ulinastatin produced direct effect right after entering into the blood. In the latter case, paraquat absorption increased with time and enhanced lung tissue damage, while ulinastatin content in blood reduced due to metabolism and lung damage gradually intensified. Compared with paraquat group, ulinastatin group still showed significantly better image changes. The magnitude of rBF, rBV decline and rPS increase were smaller and there were significant differences (P <0.05) compared with the paraquat group. The changes on the imaging suggest some treatment effect of ulinastatin.

Previous studies have shown that VEGF is a multifunctional cytokine and can regulate endothelial cell survival, proliferation, migration, angiogenesis, vascular permeability and mononuclear cell recruitment[9]. Under normal state VEGF expresses abundantly in alveolar epithelium, bronchial epithelium and bronchial gland cells and the level of VEGF in normal human respiratory alveolar fluid is 500 times more than in serum[10], but under normal circumstances it is not released directly into blood. The increasing effect of VEGF on vascular permeability is extremely strong and its effect is 20,000 times more powerful than histamine [11]. Therefore, VEGF is one of the markers to determine the degree of endothelial cell injury and vascular permeability. In this experiment, VEGF mass concentration of paraquat group elevated sharply over time. Compared with control group, there was a significant difference (P <0.05), indicating that endothelial cell damage occurs at ALI ultra-early stage and vascular permeability increases. After ulinastatin intervention, VEGF mass concentrations increased to a lesser extent. Compared with paraquat group there was significant differences (P <0.05), suggesting ulinastatin can reduce the damage of endothelial cells, inhibit the release of VEGF and reduce vascular permeability, which verified image changes from the cellular level.

Pathological observation showed that in paraquat group animals lung tissue had significant acute inflammatory changes, while ulinastatin intervention group had less inflammatory injury, which is consistent with pathology detection indicators. Pathology results also well explained the changes in the imaging and VEGF mass concentration. From comprehensive analysis, pathophysiological change at ALI ultra-early stage is vascular endothelial injury, and the exposed endothelial can easily form a local microthrombi which affects blood flow and blood volume, expressed as the decline in rBF and rBV. Endothelial injury prompts VEGF release in large quantities into the blood. The concentration, vascular permeability and rPS increase, resulting in a large number of liquid into the tissue space, alveolar septum widened and various excessive inflammatory cell infiltration to the local tissue and aggravating tissue damage. The experiments showed that early application of ulinastatin can significantly change the status of ALI ultra-early pathophysiology. It is expressed as significant improvement of vascular permeability at ALI early stage, suggesting that its mechanism should be related to reducing endothelial cell damage and lowering serum VEGF content. This is consistent with findings of Cai Shi Xia et al [12]about ulinastatin on pulmonary microvascular permeability in septic rats.

From experimental results, at ultra-early ALI stage, ulinastatin can mitigate vascular endothelial injury, reduce serum VEGF content and elevation level of pulmonary vascular permeability, lower migration and infiltration of inflammatory cells, thereby reducing the degree of lung injury and playing a role in lung protection. Studies[13,14] also showed that ulinastatin can directly inhibit the release of neutrophils and other inflammatory mediators, thereby reducing damage to the endothelial cells and reducing capillary permeability. Therefore, it is further speculated that ulinastatin on one hand can protect endothelial cells and reduce the expression of VEGF in lung tissue to inhibit neutrophil infiltration, on the other hand can directly inhibit damage of neutrophils to endothelial cell to reduce VEGF release. They promote and influence each other and the specific mechanism still needs further study.

## Competing interests

All authors declare no competing interests.

## Author contribution

ZS and GC are co-first authors. They carried out all the experiments. GL did all the data analysis work. CJ and JC prepared all the experiment materials and participated on partial of experiment work. GA designed the experiments and wrote this manuscript.

## Declarations

This article has been published as part of *BMC Emergency Medicine* Volume 13 Supplement 1, 2013: Proceedings of the 2012 Emergency Medicine Annual Congress. The full contents of the supplement are available online at http://www.biomedcentral.com/bmcemergmed/supplements/13/S1.

The publication costs for this article was funded by The People's Liberation Army No. 309 Hospital, Beijing, China.
